# Perception of dynamic facial expressions of emotion between dogs and humans

**DOI:** 10.1007/s10071-020-01348-5

**Published:** 2020-02-12

**Authors:** Catia Correia-Caeiro, Kun Guo, Daniel S. Mills

**Affiliations:** 1 School of Psychology, University of Lincoln, Lincoln, UK; 2 School of Life Sciences, University of Lincoln, Lincoln, UK

**Keywords:** Facial expressions, DogFACS, Dogs, Humans, Emotion, Face perception, Social learning

## Abstract

Facial expressions are a core component of the emotional response of social mammals. In contrast to Darwin's original proposition, expressive facial cues of emotion appear to have evolved to be species-specific. Faces trigger an automatic perceptual process, and so, inter-specific emotion perception is potentially a challenge; since observers should not try to “read” heterospecific facial expressions in the same way that they do conspecific ones. Using dynamic spontaneous facial expression stimuli, we report the first inter-species eye-tracking study on fully unrestrained participants and without pre-experiment training to maintain attention to stimuli, to compare how two different species living in the same ecological niche, humans and dogs, perceive each other’s facial expressions of emotion. Humans and dogs showed different gaze distributions when viewing the same facial expressions of either humans or dogs. Humans modulated their gaze depending on the area of interest (AOI) being examined, emotion, and species observed, but dogs modulated their gaze depending on AOI only. We also analysed if the gaze distribution was random across AOIs in both species: in humans, eye movements were not correlated with the diagnostic facial movements occurring in the emotional expression, and in dogs, there was only a partial relationship. This suggests that the scanning of facial expressions is a relatively automatic process. Thus, to read other species’ facial emotions successfully, individuals must overcome these automatic perceptual processes and employ learning strategies to appreciate the inter-species emotional repertoire.

**Electronic supplementary material:**

The online version of this article (10.1007/s10071-020-01348-5) contains supplementary material, which is available to authorized users.

## Introduction

Faces are one of the main visual channels used to convey emotional information in humans (e.g., Smith and Schyns 2009), but face-based emotion recognition (FaBER) might be quite widespread in mammals (Tate et al. 2006) due to its adaptive value. A facial expression can be an intrinsic part of the emotional response and/or a more developed social communicative action (Frijda 1986). For example, in the former case, an appropriate emotionally competent stimulus may trigger a characteristic fearful face, whereas in the latter situation, a fearful face in a social partner may be used as a prompt for flight or freezing. Being able to effectively recognise the emotional expression of another may thus confer a fitness benefit. However, inter-species emotion recognition potentially poses a challenge for individuals, as the context-specific emotional cues can be intra-specific (e.g., Caeiro et al. 2017). The human–dog dyad is an ideal model to study intra-specific perception of emotional cues, due to their shared history and ecological niche (Skoglund et al. 2015), and potential cognitive co-evolution (Hare 2007). Additionally, understanding how humans and dogs perceive each other’s facial cues of emotion has important implications for both human public safety and dog welfare.

During face exploration, humans show stereotypical gaze biases, with more fixations and longer time allocated to the eyes, followed by the nose and then the mouth (Buchan et al. 2007). This not only reflects the crucial role of the eyes in transmitting various elements of facial information, but also indicates the possible presence of a generic “hardwired” scanning strategy in the brain for general face processing (Guo 2012). Fixations on parts of the face seem to be associated with detecting and processing specific facial information, including emotion (Smith et al. 2005). Indeed, humans look relatively more at the mouth in positive emotions and at the eyes in negative emotions, presumably because these regions convey the most relevant cues for each emotion (Smith et al. 2005; Schyns et al. 2007). This has been supported by anatomically based analysis using the facial action coding system (FACS, Ekman et al. 2002a). Specifically, smiling or laughter faces display conspicuous combinations of different Action Units (AUs) in the mouth such as the lip corner puller (AU12) and the jaw drop (AU27), while fearful faces have core AUs in the eye region, such as the upper lid raiser (AU5, Ekman and Friesen 1978).

In contrast to the numerous eye-tracking studies on how humans perceive human facial expressions, research on how humans perceive dog facial expressions has mainly used relatively simple behavioural expression categorization measurements alone to largely provide clues about how humans might misinterpret facial expressions in dogs. The “guilty dog” studies (e.g., Horowitz 2009) showed humans potentially confuse dog facial responses to verbal scolding with a cognitively complex process involving a sense of guilt. From a young age, humans lack the ability to correctly interpret dogs' reactions (Meints and de Keuster 2009), and even with “information based” training in adulthood, there may be no improvement (Morrongiello et al. 2013). Even though dogs’ facial movements increase when humans are attending to them (Kaminski et al. 2017), humans may not attend to (Owczarczak-Garstecka et al. 2018) or understand (Kujala et al. 2012) relevant subtle dog signals. These results highlight the acknowledged potential communicative role perceived by humans of dog facial expressions, especially in relation to their emotional-motivational content alongside a failure to assimilate the necessary skills to do this efficiently.

Certainly, in relation to emotionally neutral dog faces, humans show a similar gaze distribution to when observing a human face (Guo et al. 2010), and this might indicate a wider use of strategies developed for human face assessment in the evaluation of the faces of other species. Although Caeiro et al. (2017) found unique relationships between certain AUs and specific emotionally competent triggers, we do not know whether any dog AU combinations are unique to specific emotional states, as occurs in humans; or to what extent humans attend to any of these AUs when dogs are emotionally aroused.

Like humans, dogs are highly attentive to human facial expressions. They can discriminate human happy expressions from neutral (Nagasawa et al. 2011), angry (Albuquerque et al. 2016) or disgusted ones (Buttelmann and Tomasello 2013; Turcsán et al. 2015); and sad from cheerful (Morisaki et al. 2009). They also show specific behavioural and physiological reactions to facial expressions. In one study (Deputte and Doll 2011), dogs avoided angry faces, and paid more attention to fearful faces, while in (Siniscalchi et al. 2018), dogs showed differential head turns to some facial expressions, and higher cardiac and behavioural activities to expressive pictures in contrast with neutral ones. Furthermore, dogs not only discriminate (e.g., Müller et al. 2015) but also recognise unfamiliar emotional expressions (Albuquerque et al. 2016) and thus must also categorise facial expressions according to their emotional content at some level. Adjusting behaviour according to the relevant emotional signals of others is biologically adaptive (Proops et al. 2018), and may be more efficient when there is an internal representation allowing the emotional classification of facial expressions.

Similar to humans, dogs prefer to fixate on internal facial features (especially the eyes, Somppi et al. 2014), but other eye-tracking studies show that fixations on facial elements depend on the facial expression, with the specifics relating to gaze allocation being somewhat inconsistent. In some studies (Barber et al. 2016; Somppi et al. 2017), dogs fixated more on the mouth and eyes in negative expressions and the forehead in positive expressions; whereas in another study (Somppi et al. 2016), dogs fixated more on the eyes and midface than the mouth in negative facial expressions and more on the eyes in pleasant faces, but attended more to the mouth of negative dog faces compared to positive ones. Somppi et al. (2017) suggest that enhanced gaze towards the eyes of emotional faces is related to the dog observer's emotional state; a perceptual bias induced by the emotional content of the face may help to focus observation on the signalling areas of greatest biological relevance (Albuquerque et al. 2018). Nonetheless, in general, dogs tend to look more towards negative dog faces than positive human faces (Somppi et al. 2016). The mixed results from these previous studies might stem from methodological issues, such as the use of static and posed facial expressions that have not been validated in terms of their content (e.g., AUs), and the use of dogs trained to stand still and to look at the screen (i.e., trained using shaping and clicker training techniques over an average of 15 sessions lasting 30–45 min each, as described in for example Karl et al. 2019) which might interfere with natural perceptual processes that require free head movements (Collewijn et al. 1992).

While these eye-tracking studies on dogs were ground-breaking in the area of dog cognition by opening a unique window into how this species perceives varied visual stimuli, we are still yet to understand the underlying specific mechanisms of facial expression perception. The recent development of more advanced eye-tracker systems (e.g., allowing movement and more data noise) allows the set-up of more naturalistic experimental conditions. Therefore, in the current study, we developed and applied a novel approach to compare human and dog perception of facial expressions: we eye-tracked participants allowing for free head (and body) movements, as they observed dynamic, spontaneous facial expressions validated by coding with human FACS and DogFACS (Waller et al. 2013). We opted to use an eye-tracking protocol that allowed fully unrestrained (i.e., the dogs were not physically manipulated or mechanically forced, sensu Alexander et al. 2011), and without specific pre-experiment fixation training (i.e., no shaping, capturing, clicker training nor any other association techniques were used to teach dogs before our experiment; for an alternative option of using an eye-tracker protocol that includes pre-training, please see Karl et al. 2019), Instead, we only used luring (i.e., a food treat is used to guide the dog into a desired position or behaviour), which focus the dog's attention on the treat, but importantly, does not create an association between reward and task (Alexander et al. 2011; Wallis et al. 2017).

We aimed to answer two main questions: (1) Where do humans and dogs naturally look when observing dynamic emotional faces and how do they compare? (2) Do humans and dogs preferentially attend to the FACS-coded facial movements? More specifically, we tested the following hypotheses: (1) Is human gaze allocation dependent on facial region, facial expression, or species observed? (2) Does human gaze allocation on facial regions differ from areas displaying AUs? (3) Does human emotion categorisation accuracy depend on species or facial expression observed? (4) Is dog gaze allocation dependent on facial region, facial expression, or species observed? (5) Does dog gaze allocation on facial regions differ from areas displaying AUs? (6) Is human and dog gaze allocation dependent on observer species, facial region, species, and facial expression observed?

## Methods

### Participants

Twenty-six human participants, between 19 and 57 years old (29.23 ± 10.35; mean ± s.d.), and 28 family pet dogs, ranging from 2 to 12 years old (5.11 ± 2.94) were recruited. One dog’s data (a Hungarian Vizsla) were discarded due to difficulty in tracking eye movements. Four humans had 1–4 missing trials and one dog had 15 missing trials due to attention or eye-tracker signal loss. More participant information is detailed in ESM 1–2.

### Experimental protocol

Testing sessions took place in a dark test room (see ESM 3 for detailed set-up configuration) at the University of Lincoln. Unlike previous studies, this study employed an eye-tracker on dogs that were fully unrestrained and without specific pre-experiment fixation training, to observe natural unconditioned responses. An Eyelink 1000 Plus eye-tracker (SR Research Ltd) in remote mode was located between the projection screen and the participant to collect the allocation of gaze on the video stimuli. The video stimuli were back-projected by an Optoma EX551 DLP projector on a semi-translucent screen (see ESM 3–5 for a video example and more protocol details).

Human participants were asked to freely identify the emotion observed after each video clip, which was recorded as the Emotion Categorisation Accuracy (ECA). For dogs, free-viewing spontaneous gaze behaviour was recorded.

### Video stimuli

Twenty videos of human and dog faces displaying four spontaneous and naturalistic facial responses to emotionally competent stimuli for fear, happiness, positive anticipation, and frustration, plus a neutral control (see Caeiro et al. 2017 and ESM 4, 5 for more stimuli details and stimuli examples) were played to the participants. Facial expressions were selected to contain the core AUs of each emotion, according to Caeiro et al. (2017). Two videos per emotion and per species were displayed. The same 20 video stimuli were played to all participants in a randomised order. To ensure that the main coder (CC) was not biased, 8 out of the 10 dog videos were coded by an independent DogFACS coder blinded to study goal and videos contexts, with 80% agreement for all AUs and ADs on the Wexler’s index (with a minimum acceptable agreement of 70%, Wexler 1972; Ekman et al. 2002b).

### Variables of interest

Areas of interest (AOIs) were drawn in the video stimuli frame-by-frame using Data Viewer 2.1.1. For humans, we defined 8 AOIs (frontal region, glabella, ears, eyes, cheeks, nose, mouth, and mental region), while for dogs, we defined 6 AOIs (nose and mouth were merged as well as ears and frontal region; see ESM 6–8 for details on AOIs definition). These AOIs were anatomically based, and thus purely hypothesis-related, and followed Hessels et al. (2016) suggestions for noise-reduction.

The primary variable of interest for this study was the viewing time, which was defined as the summation of the duration across all fixations on each AOI. Because the videos used in this study had different durations and dogs sometimes gazed at regions outside of the screen during the video presentation (see ESM 9–10 for total viewing time and respective differences), the viewing time for each AOI was normalised into the proportion of total viewing time directed at the whole face in a given video presentation (Proportion of Viewing Time—PVT). The ECA was normalised into a proportion for each category (emotion, species, etc.).

As the same AOI in faces of different species and/or different expressions varies in size (e.g., larger ears in dogs), a control for potential AOI size effect was introduced. We further calculated the probability of gazing at each AOI, termed the Likelihood of Viewing Score (LVS, adapted from Rupp and Wallen 2007; Fletcher-Watson et al. 2009): the PVT divided by the proportion of the AOI area (in pixels), i.e., the numerator is the viewing time for a given AOI divided by the total viewing time for the whole face in a given trial; and the denominator is the size of a given AOI area divided by the whole face size. The LVS allows a randomisation prediction of eye movements, i.e., if gaze allocation at each AOI is random, LVS will be close to 1. If an AOI is viewed more than chance, LVS will be larger than 1 and if viewed less than chance, LVS will take a value between zero and one (i.e., biased at or away from an AOI).

### Statistical analysis

Statistical analyses were performed with R 3.4.2. (R Core Team 2017). Data exploration and assumption checks are described in ESM 11. GLMMs with a binomial family were run for humans and dogs separately, with PVT as a response variable, AOI, emotion, and species as predictor variables, and participant number nested in case number as a random factor, using the glmer function (lme4 R-package). To investigate the LVS across the stimulus variables (AOIs, species, and emotions), one-sample Wilcoxon signed-rank tests were used with mu set at 1. If an AOI was significantly viewed more or less than chance, this was then compared with the AOIs that contained the core AUs for that emotion (Caeiro et al. 2017, ESM 7), to understand if this would bias the humans’ or dogs' gaze.

Another binomial GLMM with ECA as a response variable and PVT, emotion and species observed as predictors was run for human observers, with Kruskal–Wallis post hoc tests. Finally, to directly compare the PVT in humans and dogs, more binomial GLMMs were run for the total data set, with PVT as a response variable, AOI, stimulus emotion, stimulus species, and participant species as predictor variables, and participant number nested in case number as a random variable. Post hoc Mann–Whitney tests were then run to explore the effects of the predictor variables on PVT. To compare human and dog total viewing time, a Mann–Whitney test was also run. All models were compared using AIC (Akaike’s Information Criterion) and ANOVAs. Bonferroni corrections were applied for multiple testing based on the uncorrected α value of 0.05 for all analysis.

## Results

### Human perception of facial expressions

When modelling human PVT as the outcome, AOI, emotion and species of the stimuli were all retained in the best model (ESM 12 for details on modelling). Human face-viewing gaze allocation was first dependent on the AOI, then the viewed face species, and finally the facial expressions. Overall, the PVT was significantly lower for human faces than for dog faces, and higher for expressions featuring happiness, positive anticipation and frustration than for the neutral condition, with fear not differing from neutral. Post hoc tests showed significant differences between the PVT of all AOIs, except for the ears–cheeks, frontalis–cheeks and frontalis–ears pairwise comparisons (ESM 13).

**Fig. 1:**
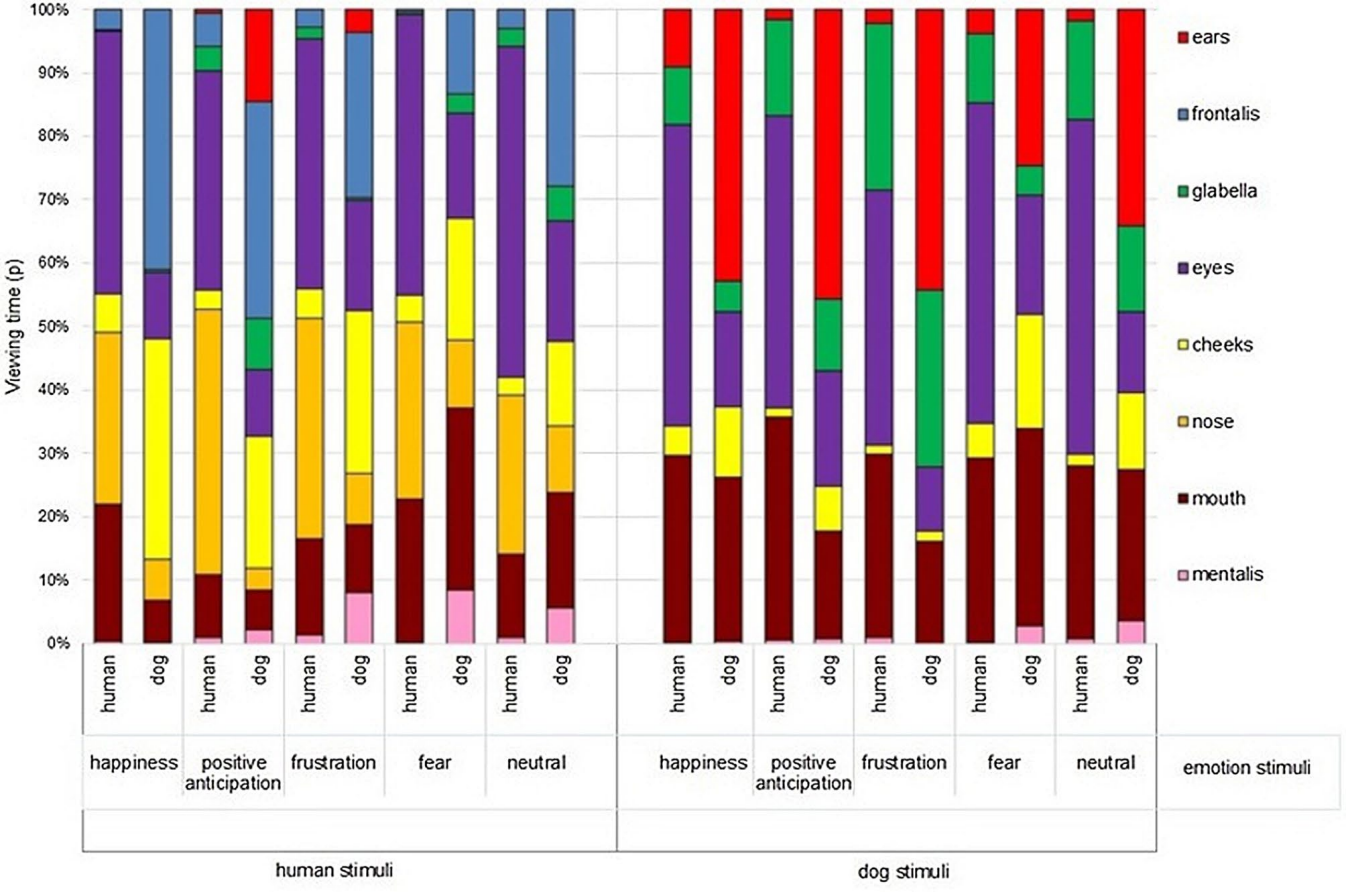
Comparison of human and dog observers’ Proportion of Viewing Time (PVT) on the face AOIs, across viewed emotions and species

When considering the data by face species and facial expressions (Fig. 1), humans looked significantly more at human eyes, nose, and mouth than other AOIs in happiness and fear, while in positive anticipation and frustration, human eyes were more viewed than the other AOIs. In neutral expressions, the eyes were significantly more viewed than the mouth, with the nose PVT falling between both, but not significantly different from either, and all three were significantly more viewed than other AOIs. When humans looked at dog faces, the eyes and mouth were similarly viewed in happiness and fear, and these were focused on more than all other AOIs. In positive anticipation and neutral faces, the eyes and mouth were again more viewed than the rest of the face, followed closely by the glabella. However, the glabella was viewed significantly less than the eyes, but not the mouth. Finally, with frustrated dogs, humans focused similarly on the eyes, mouth and glabella, and observed these more than all other AOIs (Fig. 1, ESM 14 for video example of gaze trace).

**Fig. 2:**
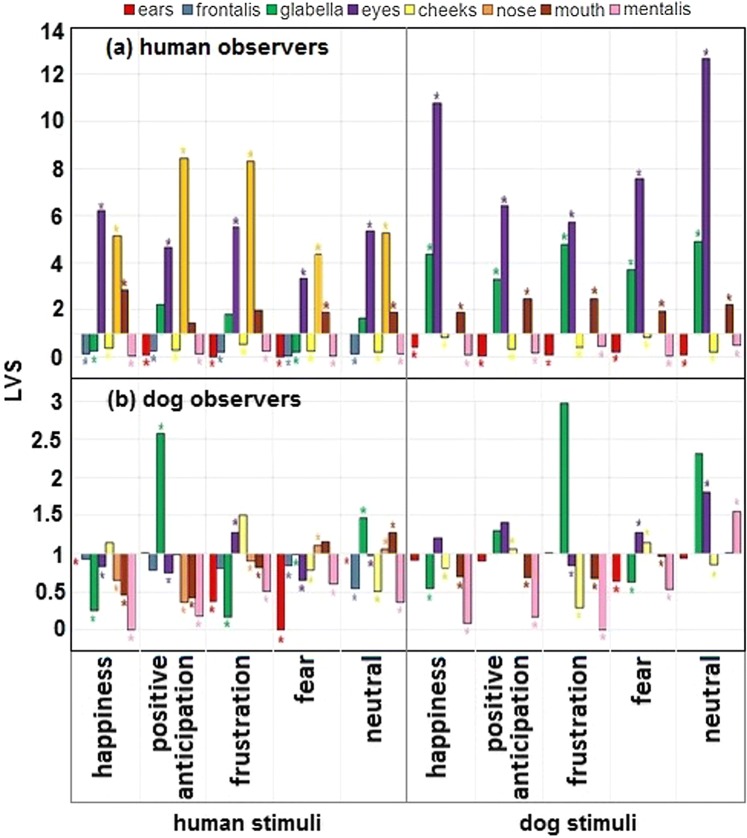
Mean likelihood of viewing time Score (LVS) in human (**a**) and dog (**b**) observers across viewed emotions and species. The further the LVS for each AOI is from 1, the more (above 1) or less (below 1) that AOI was viewed when compared to chance. Asterisk (*) indicates significance level of *P* < 0.05

When viewing human facial expressions, the LVS was not significantly different from chance for the glabella in positive anticipation, frustration, and neutral, and for the mouth in frustration and positive anticipation, while all the other tests for the AOIs in each expression were significantly different from chance (Fig. 2). The eyes and nose attracted attention much more than chance for all emotions. To a lesser extent, the mouth attracted significantly more attention in some of the emotions (happiness, fear, and neutral) than expected by chance. When viewing dog facial expressions, only the LVS on the cheeks for the fear emotion was not significantly different from chance. Humans attended to dogs’ eyes and glabella more than chance for all the emotions. Although with lower LVS, the mouth also was significantly more viewed than chance for all emotions. Furthermore, humans attended to the ears, frontalis, cheeks, and mentalis less than chance for all emotions in both human and dog faces, as well as glabella for happiness and fear in human faces (Fig. 2, ESM 15).

When modelling ECA in relation to the viewed face species and facial expressions, the best model contained only expression as an explanatory variable (ESM 14,16), indicating that when humans categorised the facial expressions of different species, the ECA varied only with the expression. Post hoc pairwise comparisons between overall ECA for each expression (ESM 17) showed all expressions to be significantly different from each other, except for happiness (0.67 ± 0.30) versus neutral (0.70 ± 0.35), both with higher accuracies than fear (0.44 ± 0.41), frustration (0.08 ± 0.18), and positive anticipation (0.06 ± 0.13). The highest ECA for human emotion was happiness (0.75 ± 0.29) and the lowest was positive anticipation (none correct), while for dog emotion, the highest ECA was for neutral (0.73 ± 0.32) and the lowest was for frustration (0.02 ± 0.10).

### Dog perception of facial expressions

When modelling dog PVT, the best model included only AOI (ESM 12), indicating that when exploring human and dog faces, the PVT of dogs varied with the face area attended, but not with the species or facial expression viewed. Post hoc tests for the AOI variable (ESM 18) showed that the mentalis region attracted significantly lower PVT than all other AOIs, while the ears and frontal region had higher PVT than all other AOIs (Fig. 1, ESM 14).

When viewing human facial expressions, the LVS was not significantly different from chance for several AOIs: the cheeks in happiness, positive anticipation, and frustration, ears in positive anticipation, eyes in positive anticipation and frustration, frontalis in happiness, positive anticipation and frustration, and, finally, mouth in fear. Dogs only directed above-chance gaze allocation at the glabella in positive anticipation and neutral, the mouth in neutral, and the nose in fear and neutral human expressions. When viewing dog facial expressions, the LVS was not different from chance level for the eyes in all expressions, the ears in all expressions except for fear, the glabella in positive anticipation, frustration, and neutral, and the mouth in neutral. Dogs only focused significantly above chance on the cheeks in the positive anticipation and fear, and the mentalis in neutral (Fig. 2, ESM 15).

### Comparison of human and dog perception of facial expressions

The best model was the full model which included all predictor variables (AOI, participant species, stimulus emotion, and stimulus species, ESM 12, 13). The PVT was first explained by the AOI, followed closely by the species of the observer, and, finally, the viewed facial expressions and face species. Overall across all AOIs, human observers (0.14 ± 0.20) had higher PVT than dog observers (0.07 ± 0.19), but dog faces (0.12 ± 0.20) were viewed longer than human faces (0.10 ± 0.19). Furthermore, all expressions (happiness: 0.10 ± 0.19, positive anticipation: 0.10 ± 0.18, frustration: 0.10 ± 0.19, fear: 0.10 ± 0.20) had significantly lower PVT than neutral (0.12 ± 0.22). The PVT between the face AOIs indicated significant differences as well (ears: 0.08 ± 0.19, frontalis: 0.08 ± 0.20, glabella: 0.06 ± 0.13, eyes: 0.24 ± 0.26, nose: 0.16 ± 0.21, cheeks: 0.06 ± 0.06, mouth: 0.15 ± 0.22, mentalis: 0.01 ± 0.07).

Post hoc analysis showed that across the viewed face species and expressions, humans, and dogs significantly differed in their PVT in most of the AOIs, except for the frontalis and mentalis that attracted similar PVT. Humans looked significantly longer at the eyes (0.42 ± 0.22), nose (0.29 ± 019), mouth (0.21 ± 0.19), glabella (0.08 ± 0.12) and cheeks (0.03 ± 0.06) than dogs (mouth: 0.09 ± 0.23, eyes: 0.07 ± 0.17, glabella: 0.04 ± 0.13, nose: 0.04 ± 0.14), while dogs viewed significantly more the ears (0.14 ± 0.25) and cheeks (0.08 ± 0.20) than human observers (ears: 0.02 ± 0.05, cheeks: 0.03 ± 0.06).

Significant differences were also found between human and dog PVT towards the AOIs, when split by the viewed face species (ESM 19). Human observers attended significantly less to other humans’ glabella than dogs, but more to other humans’ eyes, nose, and mouth. When looking at dog faces, humans looked more at the glabella, eyes, and mouth than dogs, while dogs focused more than human observers on the ears and cheeks.

Furthermore, humans and dogs exhibited significant differences in PVT at the same AOI for human facial expressions (Fig. 1, ESM 13), with humans showing a higher PVT for the eyes, nose, and mouth in all expressions, and glabella in frustration, when compared with dogs’ PVT. On the other hand, dogs attended significantly more to the human glabella in positive anticipation than human observers. Likewise, when observing the AOIs of dog faces, humans focused significantly more on the glabella, eyes, and mouth of dogs in all emotional contexts than dog observers.

## Discussion

### Human perception of facial expressions

Our observations in relation to the first hypothesis agree with the previous research, where the human gaze was modulated by facial region, expression, and species observed. There was a clear gaze allocation bias towards the eyes in human face-viewing, followed by the nose and then the mouth (Henderson et al. 2005; Buchan et al. 2007; Guo et al. 2010), with the mentalis as the least attended AOI. This is believed to be a relatively ‘hardwired’ mechanism (Tate et al. 2006; Guo 2012), with the eyes providing a wealth of valuable information, ranging from ostensive cues (e.g., eye contact with eyebrow flash to initiate interaction: Eibl-Eibesfeldt 1972) to subtle and/or implicit cues (e.g., saccades as deception cues Vrij et al. 2015). Interestingly, although the eyes and nose tended to attract a well-above-chance level of viewing time regardless of expressions, the results addressing our second hypothesis showed that they did not always match the location of the expression-specific dynamic information identified by FACS, i.e., humans did not always attend to the regions displaying the most relevant AUs (ESM 8). Furthermore, the mouth, followed by the eyes and nose, had increased PVT in happiness and fear (also found in, e.g., Eisenbarth and Alpers 2011), which only partly matches the diagnostic AUs displayed, since the eyes are more important than the mouth AUs in both (genuine) happiness (AU6) and fear (AU5). This is likely due to the human tendency to follow a pre-determined face-scanning pattern to process any facial expression, without attention bias to informative dynamic AOIs. Since processing of these cues cannot be done until the fovea reaches the informative AOI, the hardwired scanning process is activated instead, with the PVT adjusted as needed. For example, in happiness, the gaze starts on the eyes, moves to the nose, and finally mouth where it lingers, instead of starting on the mouth (i.e., the most conspicuous AUs), and then moving to the eyes (i.e., the diagnostic AU). Further support for a hardwired face-scanning pattern comes from the human viewing of dog faces, which was mostly directed towards the dog’s eyes and mouth (+ nose). This too does not match the FACS-coded AUs with potential emotional content of the facial expression. For example, humans focused more on dogs’ eyes and mouth in happiness, whereas its core AU appears only on the mouth (Caeiro et al. 2017). This is consistent with the finding that similar brain activation occurs when viewing the faces of both species (Blonder et al. 2004); i.e., it seems that a human-like facial search strategy is used to perceive dog faces.

Interestingly, dog frustration and positive anticipation also attracted a gaze bias towards the glabella. The glabella in dogs includes the AU101—inner brow raiser, which is attractive to human observers (Waller et al. 2013). Hence, humans were likely concentrating on the eyes and surrounding regions, such as the glabella, in more ambiguous or hard to detect emotions. Human positive anticipation does not have a prototypical facial expression and cannot be identified by the face alone (Rosenberg and Ekman 1997) as also shown by the null ECA. Dog frustration faces also had the lowest ECA, which indicates that human observers were not looking at informative areas per se, but instead were deploying the hardwired scanning strategy centralised on the eyes. The results relating to our third hypothesis (i.e., testing how accurate humans were at categorising emotions in human and dog faces) also indicated that emotion identification is dependent on emotion category, where some emotions are harder to identify, both in humans and dogs (e.g., frustration).

In summary, humans failed to attend to especially relevant dog AUs, with the striking example of the ears, a highly mobile feature (Waller et al. 2013) central in dog communication, being less observed in all emotions. Our results may thus explain why humans struggle to identify dog emotion accurately: their hardwired human facial expression recognition strategy is not adapted for the most informative facial areas of heterospecifics. Even with training (Schwebel et al. 2012), humans seem to have great difficulty in cross-species perception, which suggests that social learning strategies might also fail.

### Dog perception of facial expressions

The results addressing our fourth hypothesis showed dogs' face-viewing gaze allocation varied between AOIs, but not between species or expressions observed, suggesting that their viewing strategy is even more rigid than that of humans. Dogs looked more often at the ears, mouth, and eyes in dog faces, and the frontalis, eyes, nose, and cheeks in human faces. Previous studies have had mixed results regarding preference for own- *vs* other-face species (own: Somppi et al. 2012, 2014, 2016); human: Törnqvist et al. 2015), and particular emotional expressions: (Racca et al. 2012; only with species–emotion interaction: Somppi et al. 2016). These conflicting results may stem from methodology (e.g., visual paired comparison *vs* eye-tracker, static *vs* dynamic stimuli). Our results are in contrast with one potentially comparable study using static images (Kis et al. 2017), where the human eyes and mouth were attended to more, but partially agree with Barber et al. (2016) where the human mouth and cheeks + nose (i.e., AOI “face rest”) were fixated on more than the rest of the face. Kis et al. (2017) argued that the forehead was less informative, which appears inconsistent from both an anatomical and communicative stance (e.g., the eyebrow flash, Eibl-Eibesfeldt 1972; or expression of fear, Ekman et al. 2002b). The frontalis muscle produces conspicuous changes on the forehead (Ekman et al. 2002a), by pulling the eyebrows and hairline upwards, and wrinkling the forehead (AU1 + AU2). However, this dynamic information, which impacts gaze allocation (Buchan et al. 2007), neural pathways (Kilts et al. 2003) and meaning (Schyns et al. 2007), is absent in static images. Nonetheless, Barber et al. (2016) found the human forehead to be the most attended in positive expressions, while eye and mouth were more fixated in negative expressions. Other studies (Somppi et al. 2014, 2016) reported dogs looking longer at the eyes in both species and all emotions, with the mouth of threatening dogs attended to more than of neutral or pleasant dogs, or of threatening humans (Somppi et al. 2016). Unfortunately in these studies, the ears and frontal region of dogs were not analysed, despite its importance in canid behaviour. Nevertheless, static images potentially give misleading information about the natural viewing patterns deployed for evaluating emotional faces.

Another methodological concern is the difficulty in operationally defining comparable AOIs for two morphologically very different species, as in Somppi et al. (2016) where the “mouth” AOI in dogs excludes the upper lip and includes the mental region, while in the human stimuli, the chin was excluded. Furthermore, without full control of the content of the image in terms of AUs, it is likely that strongly biased stimuli sets towards how humans perceive facial expressions through anthropocentric and/or holistic emotion processing may be used, rendering it difficult to interpret the results from an independent ecological perspective. Nonetheless, these previous studies were ground-breaking in using eye-tracking on dogs to investigate their perceptual world, particularly regarding emotional content of stimuli.

The lower attention towards the eyes from dog observers might alternatively (or additionally) stem from the functional significance of eye contact in dogs compared to humans. While humans engage in prolonged eye gaze for mostly positive reasons (e.g., emotion perception, communicative intent, Senju and Csibra 2008; Kis et al. 2017), with lack of eye contact interpreted negatively (Larsen and Shackelford 1996), in canids, and many other species, a fixed stare is linked to agonistic contexts (McGreevy et al. 2012; Kis et al. 2017).

Dogs’ gaze did not differ with the emotion or the species observed, and focused more on the ears and frontalis of all individuals observed, followed by the mouth. This is an important difference from human gaze behaviour and may reflect the significance given to the ears, frontalis, and mouth as potentially informative regions for dogs. Indeed, ears and mouth in dogs have been found to display diagnostic AUs for happiness, positive anticipation, and fear (Caeiro et al. 2017). However, we cannot be sure if they are looking more at the frontalis, because it is an informative region in humans and potentially dogs (as discussed above) or if it stems from direct eye contact avoidance. Ears in humans are generally considered to be information irrelevant, so it is not clear why dogs look at human ears, unless it is part of a fixed scanning process.

In relation to our fifth hypothesis, dogs only attended to the human nose more than expected with the fear stimuli. The nose in fearful human faces produces a very small movement as a core AU (AU38—Nostril dilator), suggesting that dogs attend to very subtle movements. This movement is also present in dogs (during sniffing, AD40), and so AU38 might be a cue important in both species. However, dogs also looked at the nose more than expected in the neutral condition, so another explanation for this result is that the nose is a central point between eyes and mouth cues (both relevant in human fearful faces), facilitating general face exploration. In support of this explanation, dogs did not attend especially to areas with facial movement, performing instead a more general scanning of the whole face of humans (glabella, mouth, and nose) and the lower face of dogs (cheeks and mentalis). It is unlikely that dogs simply are working hard to attend to all the facial elements to look for information, because, if this was the case, the areas of the face attended to should reflect the information available from these regions in any given state. Instead, the configural processing of familiar faces in dogs (Pitteri et al. 2014) is here extended to facial expressions. Configural processing (sensu Bruce and Young 2012) is early and well developed in humans (de Heering et al. 2007), and found in varied taxa, including chimpanzees (Parr et al. 2008), sheep (Kendrick et al. 1996), pandas (Li et al. 2017), and even bees (Dyer 2005). Therefore, if this is an ancient mechanism in animals, which is subjected to perceptual narrowing with stimulus familiarity (Sugita 2008), we suggest that dogs employ the same mechanism under a social learning strategy for cross-species emotion perception.

### Comparison of human and dog perception of facial expressions

The results for our last hypothesis tested demonstrated that gaze allocation was dependent of the observer species, and the facial regions, species, and expressions observed. Humans had higher PVT for all stimuli compared to dogs. Human observers focused more than dog observers on human eyes, nose, and mouth, and on dog glabella, eyes, and mouth for all emotions. On the other hand, dog observers focused more than human observers on the human glabella, and on the dog ears and cheeks. These results highlight markedly different visual inspection strategies between the two species when observing expressions of emotion.

Only three studies to date have compared human and dog perception of own *vs* other species (Guo et al. 2009; Racca et al. 2012; Törnqvist et al. 2015); all support our results of a lower PVT for all stimuli by dogs, i.e., dogs were quicker than humans, regardless of stimuli or method used. It might be argued that dogs have shorter attention spans (although this has not yet been investigated), are more easily distracted/bored (Burn 2017), or tend to avoid fixed stares, but this is unlikely, since the previous studies have reinforced the dogs to look at the stimuli. Another plausible explanation is that dogs simply have quicker processing mechanisms in general. Human facial muscles contract slower than other species (Burrows et al. 2014), while dogs have more mobile and quicker facial muscles than wolves (Burrows et al. 2017; Kaminski et al. 2019). If dog facial cues are quicker than in human faces, it would be reasonable to assume that conspecific observers need less time to decode these cues. Additionally, humans likely extract additional information from a face (attractiveness, age, etc.) and use higher cognitive functions, slowing overall processing, while dogs might be more efficient by aiming at the most biological relevant facial information, i.e., emotion. Interestingly, the same phenomena are also found in apes (Kano et al. 2012), who surpass humans in both speed and accuracy in visual cognitive tasks (e.g., memory: Inoue and Matsuzawa 2007).

Intra-specific perception of emotion is rapid and automatic, occurring via a phylogenetically ancient subcortical route, independent of conscious awareness (Johnson 2005; Adolphs 2006); however, in mammals, there is also a cortical route, allowing more flexible behaviours based on learning and conscious appraisals (Adolphs 2006). Accordingly, the automatic mechanism for conspecific facial perception may need to be adapted through cortical learning for the efficient decoding of heterospecific facial expressions of emotion, given its species specificity (Caeiro et al. 2017). Indeed, it seems that humans do not naturally learn to look at the relevant AUs for each expression in dogs; by contrast, pet dogs appear to do this partially in relation to human faces. Therefore, we suggest that efficient heterospecific cue processing involves more than learning the other species repertoire, requiring also learning to suppress automatic neural responses to cues associated with the processing of the emotional content of conspecific faces. Thus individuals need to learn not only where to look, but also to suppress own species-specific biases when evaluating relevant cues.

## Summary and future directions

By building upon those pioneer dog eye-tracking studies (e.g., Somppi et al 2012, 2016), our work aimed at further investigating perceptual mechanisms underlying facial expression perception in dogs. We demonstrated that humans and dogs observe facial expressions of emotion by employing different gaze strategies and attending to different areas of the face, with humans' strategies dependent on species and expression observed, while dogs maintain the same pattern regardless of stimulus class. These differences may be largely driven by automatic processes adapted for conspecifics. More importantly, the facial areas attended by humans often do not overlap with the regions where AUs are displayed, and in dogs they only do partially. Hence, facial movements do not attract attention, suggesting that intra-specific hardwired processes dominate face-viewing.

Future research could examine the other aspects of eye movements in dogs’ face-viewing behaviour, such as scan paths. The order and repetition of fixations in particular facial regions can provide further clues on dogs’ priority in processing different local expressive facial cues. The testing protocol could also be further improved. In this study, we recorded naturalistic and spontaneous face-viewing behaviour in dogs without specific pre-experiment training (e.g., without shaping, clicker training and other associative techniques before the start of the experiment, as opposed to for example in Karl et al. 2019) or any specific fixation on stimuli training; we used only luring (sensu Alexander et al. 2011; Wallis et al. 2017) to focus the dog's attention on a treat, to, for example, guide the dog into position and attend to the drift points on the screen, but not to create an association between task and reward. This methodological choice is important, because we aimed at studying naturalistic emotional responses in dogs, which might be impacted if extensive and/or intensive training is used before the experiment. While we did not reinforce sustained attention, we used treats or toys for luring the individuals throughout the protocol, which might not be appropriate for all research questions (e.g., fMRI studies, Karl et al. 2019). Furthermore, we used a protocol that allowed small head/body movements from unrestrained dog participants that were not physically manipulated or mechanically forced into a particular position, which leads to more spontaneous responses. However, this protocol is also susceptible to head/body large movements and has led to less data being collected. In the future, it is important to consider the type of research question being asked and the best protocol to address it, given the diversity of methods now reported in the literature.

Nevertheless, our work presents an alternative eye-tracking protocol using only luring, and, more importantly, has fundamental implications for our understanding of both the theoretical underpinnings of inter-specific emotional communication, as well as within the human–dog relationship, where the emotional state of the other species, is commonly (mis)perceived.

## Electronic supplementary material

Below is the link to the electronic supplementary material.

Supplementary file1 (DOCX 346 kb)

Supplementary file2 (MP4 50844 kb)

Supplementary file3 (MP4 45041 kb)

Supplementary file4 (XLSX 780 kb)
