# Supplementary material for: Perception of dynamic facial expressions of emotion between dogs and humans
Source: Anim Cogn. 2020 Feb 12;23(3):465–76. doi: 10.1007/s10071-020-01348-5 (PMC7181561; doi:10.1007/s10071-020-01348-5)
Supplement: Supplementary file 1 — Supplementary file1 (DOCX 346 kb) [file 10071_2020_1348_MOESM1_ESM.docx]

Perception of dynamic facial expressions of emotion between dogs and humans

Catia Correia Caeiro^1,2*^; Kun Guo^1^; Daniel Mills^2^

^1^School of Psychology, University of Lincoln, UK.

^2^School of Life Sciences, University of Lincoln, UK.

***Corresponding author**: Catia Correia Caeiro, ccorreaicaeiro@lincoln.ac.uk

# Electronic Supplementary Material

**ESM 1:** Recruitment of both human and dog participants was done through the University News page, local social media groups and local businesses. Dogs were also recruited through the University database LincolnPetsCanDo (<http://www.lincolnpetscando.co.uk/>). Human participants and dog owners received a £5 voucher after study completion. The only exclusion criterion for humans was known uncorrected visual deficits. Exclusion criteria for dogs were known or overt vision issues or aggressive behaviour towards unfamiliar people.

**ESM 2:** List of human (with age, gender and cultural group) and dog (with age, sex and breed) participants:

| Human participant # | Age (years) | Gender | Cultural group |
| --- | --- | --- | --- |
| 1 | 21 | male | British white |
| 2 | 21 | female | European white |
| 3 | 29 | female | British white |
| 4 | 19 | female | British white |
| 5 | 20 | male | British white |
| 6 | 38 | female | British white |
| 7 | 22 | female | British white |
| 8 | 26 | male | British white |
| 9 | 37 | male | British white |
| 10 | 37 | female | British white |
| 11 | 20 | male | British white |
| 12 | 20 | female | British white |
| 13 | 19 | female | British white |
| 14 | 23 | male | British white |
| 15 | 33 | female | European white |
| 16 | 57 | female | British white |
| 17 | 30 | female | British white |
| 18 | 43 | female | British white |
| 19 | 26 | male | British white |
| 20 | 26 | female | British white |
| 21 | 43 | male | British white |
| 22 | 31 | female | British white |
| 23 | 28 | female | European oriental mixed |
| 24 | 50 | female | British white |
| 25 | 22 | female | British white |
| 26 | 19 | female | British white |

| Dog participant # | Age (years) | Sex | Breed |
| --- | --- | --- | --- |
| 1 | 9 | male | Border Collie |
| 2 | 2 | male | Border Collie |
| 3 | 5 | female | Mixed |
| 4 | 3 | male | Hungarian Vizla |
| 5 | 2 | female | Mixed |
| 6 | 7 | male | Jack Russell |
| 7 | 3 | female | Large Munsterland |
| 8 | 10 | male | Golden Retriever |
| 9 | 3 | female | Cocker Spaniel |
| 10 | 7 | female | German Shorthaired Pointer |
| 11 | 3 | male | Mixed |
| 12 | 12 | male | Golden Retriever |
| 13 | 4 | female | Cocker Spaniel |
| 14 | 2 | male | Border Terrier |
| 15 | 4 | female | Labrador Retriever |
| 16 | 9 | female | Labrador Retriever |
| 17 | 2 | male | Mixed |
| 18 | 6 | female | Labrador Retriever |
| 19 | 4 | male | Labrador Retriever |
| 20 | 5 | male | Labrador Retriever |
| 21 | 5 | male | Chihuahua |
| 22 | 4 | male | Mixed |
| 23 | 4 | male | Mixed |
| 24 | 2 | female | Smooth Collie |
| 25 | 3 | male | Duck Tolling Retriever |
| 26 | 7 | male | Labrador Retriever |
| 27 | 11 | male | Labrador Retriever |
| 28 | 9 | male | Border Collie |

**ESM 3**: **Test room diagram**: A – Cameras synchroniser and screen; B – Eye-tracker Host Laptop; B1 – 2nd screen of eye-tracker host laptop; C – Projector, display computer; D – Eye-tracker camera; E – Video cameras; F – Screen, G – Window frame, H - Maximum width of stimuli, I - Water bowl, 1 – Experimenter 1 (not visible to participant), 2 – Experimenter 2 (back visible to participant):


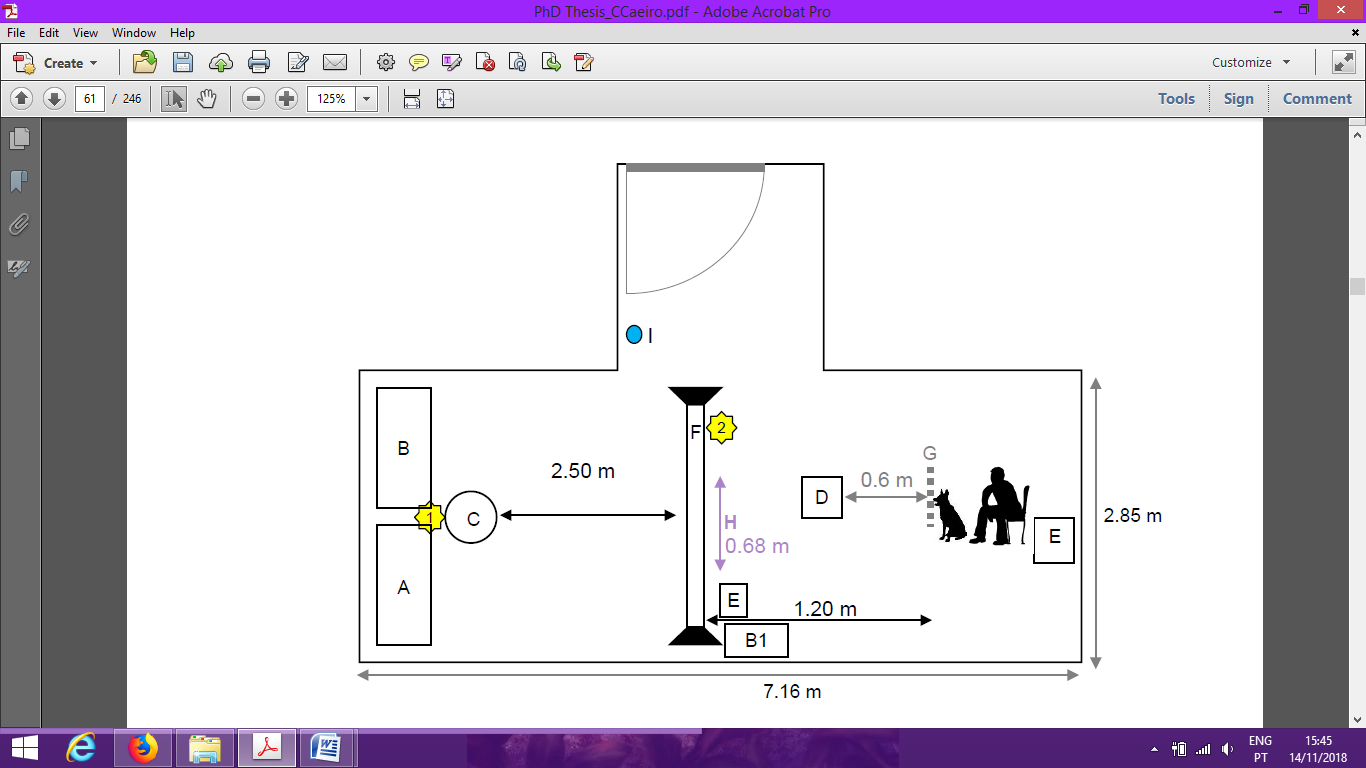


**ESM4:** See video file.

**ESM 5: Protocol and video stimuli details:** The Eyelink eye-tracker (500Hz sampling frequency, 0.25–0.5° accuracy and 0.01° root-mean-square resolution) was placed 60cm away from both screen and participant, slightly off-centre, in order to be able to track the dog’s eye without the nose blocking the camera view or the IR light. The screen (185cm×140cm, 88.67°×66.35°) was placed between the participant and the experimenter controlling the eye-tracker, in order to avoid any unconscious cues from the experimenter. The dog participants were lured with a treat or toy to the mat behind the window frame and allowed to spontaneously chose to sat, stood or laid down in front of the owner (or an assistant, if the owner chose to not attend the session). The owner/assistant did not restrain or positioned the dog in any particular way (i.e. did not physically manipulated nor mechanically forced the dog, *sensu* Alexander et al. 2011). No chin/head rest was used, as the dog's head could freely move behind the window frame. The dog was free to choose how to position itself behind the window frame (determined during calibration) and it was free to leave at any point. If the dog chose to leave, the display of the next stimulus would be paused, the experimenter would wait 1-2 min (e.g. for the dog to drink water, walk around the room, etc.) and then call the dog or lure the dog with a treat to come behind the window frame again. Treats would be used to lure (i.e. guide/encourage, *sensu* Alexander et al. 2011, Wallis et al. 2017) the dog to get back behind the frame.

Calibration and validation were performed for each participant with the Eyelink built-in 3-point calibration [(0°, -18.2°), (-25.9°,18.2°), (25.9°,18.2°)] for dogs, and 5-point [(0°,0°), (0°,18.2°), (0°,-18.2°), (-25.9°,0°), (25.9°,0°)] for humans. A second experimenter held a treat or a small toy in front of the white calibration or drift point (0.8° in diameter) to ensure dogs were fixating on the desired point on the screen, prior to stimulus presentation. Dynamic drift points were used for dogs (bouncing ball) and static drift points identical to calibration points for humans, randomly placed on each side of the screen [(-11.5°,0°), (11.5°,0°)] before each new stimulus to minimise the central fixation bias. Calibration points and stimuli were displayed against a grey background. Both species were required to fixate the point for 1 sec before acceptance.

The temporal interval between video display (inter-trial interval) was variable due to the manual drift point correction procedure (drift points have several important functions, including correcting for large head movements between videos, avoiding central biases, standardising the first fixation on the screen and refocusing dog's attention). In our study, the drift point was manually recorded because the dogs were not trained specifically to look at the screen, hence the interval would last as long as the dog took to focus an eye for at least 1 sec on the target. After each video, the dogs were given a treat, regardless of their behavioural responses or viewing behaviour to the video, i.e. regardless of whether they watched the video or any particular area of the screen. The experimenter giving the treat to the dog could not see the behaviour of the dog during stimulus display. Thus there was no specific behavioural reinforcement in relation to the facial stimuli presented. The treat was used mainly to keep the dog interested and to focus their attention on the drift points. If the dog did not watch more than 50% of the videos (confirmed by looking at the eye-tracking data and video recording of the individual, after finishing the display), the dogs would be played the stimuli again after a break (~15 min).

Each of the 20 video stimuli ranged from 5-12 sec, where variation in duration was due to inherent variation in the display of spontaneous naturalistic emotional expressions. Each video was edited to contain just the face, with all the AOIs visible, and standardised by face height to display within vertical calibration points (36°), at 30 fps. All videos had different individuals and included adult men, women and dogs extracted from research databases or YouTube (see [4] for more information on video selection). We ensured there was no Flicker-Fusion Frequency effects between the two species tested by using a DLP projector without flickering (light output is constant).

**ESM 6**: Areas of Interest (AOI) for both humans and dogs based on the corresponding musculature and facial units from Human FACS and Dog FACS. ✔- Present/defined, 🗙 – absent/not defined, H – humans, D – dogs, AUs – Action Units, ADs – Action Descriptors, EADs – Ear Action Descriptors, m. – muscle:

| **AOI label** | **Species AOI** | | **Muscles present in the AOI** | | **AUs/ADs/EADs** | | **Notes** | **References** |
| --- | --- | --- | --- | --- | --- | --- | --- | --- |
|  | **H** | **D** | **H** | **D** | **H** | **D** |  |  |
| Ears | ✔ | ✔ | Superior/ anterior auricular and postauricular m. Vestigial m., limited movement in some individuals | Set of complex and well developed muscles, including occipitalis, parotidoauricularis, cervicoauricularis, cervicoauricularis superficialis, cervicoscutularis, interscutularis. | 🗙 | EAD101 – Ears forward  EAD102 – Ears adductor  EAD103 – Ears flattener  EAD104 – Ears rotator  EAD105 – Ears downward |  | Alvord & Farmer 1997, Benning 2011, Burrows et al 2017, Adams 2004, Smith 1999, Done et al 1996, Miller et al 1964 |
| Frontal region | ✔ | 🗙 | Frontalis (medial and lateral) | Frontalis is present but no movement was identified in the frontal region connected to this muscle. | AU1 – Inner brow raiser  AU2 – Outer brow raiser | 🗙 | As most of the movement in the frontal region of dogs is from the ears, the frontal region was included in the Ears AOI. | Ekman et al 1978, 2002, Waller et al 2013, Waller et al 2013 |
| Glabella | ✔ | ✔ | Procerus, corrugator supercilii, depressor supercilii | Levator anguli occuli medialis raises the inner brow saliency in dogs. | AU4 – Brow lowerer | AU101 – Inner brow raiser | AU101 produces movement in the glabella region by pulling the upper eyelid upwards. |  |
| Eyes | ✔ | ✔ | Orbicularis occuli, levator palpebrae | Orbicularis occuli, retractor anguli occuli lateralis | AU43 – Eye closure  AU45 – Blink  AU6 – Cheek raiser | AU143 – Eye closure  AU145 - Blink | AU6 causes visible movement on the face by bulging/raising the cheeks and by wrinkling the outer eye corners. |  |
| Cheeks | ✔ | ✔ | Orbicularis occuli, zygomaticus major/minor | Orbicularis occuli, zygomaticus | AU6 – Cheek raiser | Observed with AU143 and AU145 |  |  |
| Nose | ✔ | 🗙 | Levator labii superioris alaeque nasi, nasalis | Levator nasolabilais, caninus, levator labii maxillaris, zygomaticus, orbicularis oris, buccinator | AU9 – Nose wrinkler  AU38/AU39 – Nostril dilator and compressor | AU109+110 – Nose wrinkler and upper lip raiser  AD40 - Sniff | Nose movements in dogs are not independent so the AOI nose and mouth are merged. |  |
| Mouth | ✔ | ✔ | Levator labii superioris, zygomaticus major/minor, caninus, buccinators, depressor anguli oris, depressor labii inferioris, incisivii labii, orbicularis oris |  | AU110 – Upper lip raiser  AU11 – Nasiolabial furrow deepener  AU12 Lip corner puller  AU13 – Sharp lip puller  AU14 – Dimpler  AU15 – Lip corner depressor  AU16 – Lower lip depressor  AU18 – Lip pucker  AU20 – Lip stretcher  AU22 – Lip funneler  AU23 - Lip tightener  AU24 – Lip Presser  AU25 – Lips Part  AU26 – Jaw drop  AU27 – Mouth stretch | AU110 – Upper lip raiser  AU12 – Lip corner puller  AU116 – Lower lip depressor  AU118 – Lip pucker  AU25 – Lips part  AU26 – Jaw drop  AU27 – Mouth stretch |  |  |
| Mental region | ✔ | ✔ | Mentalis | Mentalis | AU17 – Chin raiser | 🗙 |  |  |

**ESM 7**: Table of correspondence between the emotional contexts of the video stimuli, the facial movements present in each emotion and the AOI that includes the facial movement (Caeiro et al 2017). For example, during happy videos of humans, the core facial movement is caused by AU6 which is included in the Eyes and Cheeks AOIs:

| **Emotion** | **AUs/ADs/EADs** | | **AOI** | |
| --- | --- | --- | --- | --- |
|  | **H** | **D** | **H** | **D** |
| Happiness | AU6 – Cheek raiser | AU27 – Mouth stretch | Eyes  Cheeks | Mouth |
| Positive Anticipation | - | AD37 – Lip wipe  AD137 – Nose lick  EAD102 – Ears adductor | - | Mouth  Ears |
| Frustration | AU14 - Dimpler  AU17 – Chin raiser  AU24 – Lip presser  AU28 – Lips suck  AD84 - Head shake back and forth | - | Mouth | - |
| Fear | AU5 – Upper eyelid raiser  AU7 – Lids tightener  AU20 – Lip stretch  AU38 – Nostril dilator | AD126 - Panting | Eyes  Mouth  Nose | Mouth |
| Neutral | AU0 | AU0 | - | - |

**ESM 7.1:** The AOIs in this study were defined according to established anatomical knowledge of facial muscle insertion and attachment for both dogs and humans as well as the corresponding AUs for each AOI (see also **ESM 6**). Due to the different morphological configuration of human and dog faces, slightly different AOIs were defined. These are not based on perceptual human inferences, but are instead grounded on the external morphology (e.g. the nose is located in front of the mouth in dogs, while in humans the nose is located above the mouth) and the internal facial morphology, i.e., the underlying musculature and the movement each region produces (e.g. the muscles producing movement around the nose are the same as the mouth in dogs, while in humans they are independent).

**ESM 8:** Example of AOIs in the human and dog face stimuli, with respective AOI labels (see also ESM 6):


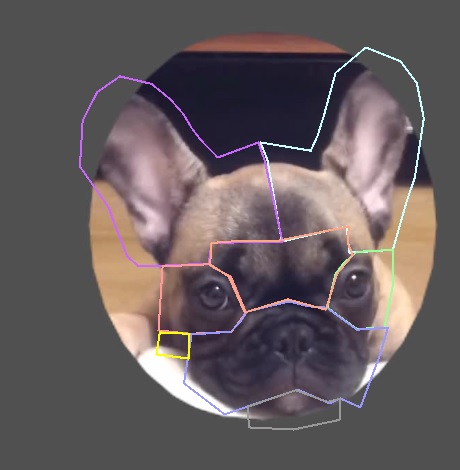

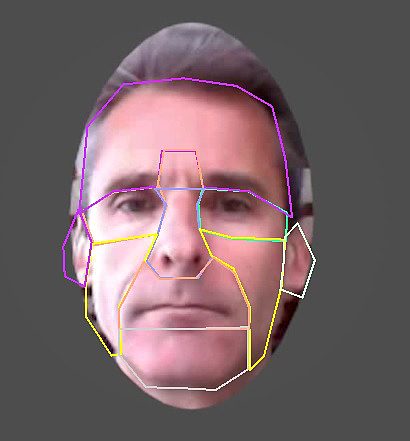


Frontal region

Glabella

Ears

Eyes

Nose

Mental region

Mouth

Cheeks

**ESM 9:** Mean and standard deviation of the proportion of total viewing time of the stimuli (AOI window) for the dog and human observers:

|  |  | **Participant species** | | | |
| --- | --- | --- | --- | --- | --- |
| **Stimulus species** |  | **Dog** | | **Human** | |
|  | **Stimulus emotion** | **MEAN** | **SD** | **MEAN** | **SD** |
| Human | Happiness | 0.2101 | 0.2708 | 0.8420 | 0.1251 |
|  | Positive anticipation | 0.2260 | 0.2644 | 0.8539 | 0.0803 |
|  | Fear | 0.2573 | 0.2733 | 0.8342 | 0.0992 |
|  | Frustration | 0.3388 | 0.2599 | 0.8596 | 0.0558 |
|  | Neutral | 0.1543 | 0.2609 | 0.8587 | 0.0802 |
| **Subtotal** |  | 0.2382 | 0.2708 | 0.8497 | 0.0909 |
| Dog | Happiness | 0.3251 | 0.2669 | 0.8456 | 0.0648 |
|  | Positive anticipation | 0.3475 | 0.2955 | 0.8287 | 0.1432 |
|  | Fear | 0.3401 | 0.3309 | 0.8391 | 0.1274 |
|  | Frustration | 0.3440 | 0.3240 | 0.8611 | 0.0728 |
|  | Neutral | 0.3539 | 0.3327 | 0.8580 | 0.0636 |
| **Subtotal** |  | 0.3421 | 0.3087 | 0.8466 | 0.1002 |
| **Total** |  | 0.2908 | 0.2949 | 0.8481 | 0.0956 |

**ESM10:** Mann-Whitney results comparing the proportion of total viewing time of the stimuli (AOI window) for the dog and human observers:

|  | **U** | **p** | **SE** |
| --- | --- | --- | --- |
| **Total viewing time** | 249735 | .0001 | 4799.453 |

**ESM 11:** Data exploration and assumption checks: As it is not yet known what a typical eye movement pattern in dogs as a species looks like, no outliers were removed from the data. Data exploration was performed with Cleveland plots and boxplots for each data subset, separated by group of interest (e.g. AOI). Normality was verified with histograms with normal curve and Shapiro-Wilk tests. Overdispersion checks were performed by comparing the full model residual variance and the degrees of freedom. Multicollinearity of variables was tested using the VIF function (car R-package).

**ESM 12:** GLMM comparisons additional information, with AIC values and post-hoc tests:

All data were non-normal, but did not present overdispersion or collinearity between variables.

**12.1.Human data PVT GLMM**: The largest rise in the AIC was seen when AOI was dropped from the model (full model AIC: 38909.4 *vs* model dropping AOI: 41608.8, ANOVA: *F_1_*=2713.4, *p*=.0001), which indicated this variable as impacting the response variable the most. Emotion and species were also significant in explaining the data in the model, as when either were dropped, the AIC would significantly rise, with species rising slightly more when dropped from the model (full model AIC: 38909.4 *vs* model dropping emotion: 38925.9, ANOVA: *F_1_*=24.481, *p*=.0001; and *vs* model dropping species AIC: 39085.7, ANOVA: *F_1_*=178.32, *p*=.0001).

Best GLMM model for PVT as a response variable and the predictor variables AOI, stimulus emotion and stimulus species for the human subset data:

| **Predictor variables** | **Estimate** | **SE** | **z** | **p** |
| --- | --- | --- | --- | --- |
| Response variable: PVT |  |  |  |  |
| Intercept | -7.0664 | 0.2713 | -26.04 | 0.0001 |
| AOI (Ears) | -2.1831 | 0.3248 | -6.72 | 0.0001 |
| AOI (Eyes) | 7.1379 | 0.2699 | 26.44 | 0.0001 |
| AOI (Frontalis) | -1.0332 | 0.3781 | -2.73 | 0.0063 |
| AOI (Glabella) | 1.2900 | 0.2779 | 4.64 | 0.0001 |
| AOI (Mentalis) | -5.7883 | 0.3034 | -19.08 | 0.0001 |
| AOI (Mouth) | 5.1877 | 0.2700 | 19.21 | 0.0001 |
| AOI (Nose) | 7.5040 | 0.3342 | 22.45 | 0.0001 |
| Stimulus emotion (Happiness) | 0.9122 | 0.2414 | 3.78 | 0.0002 |
| Stimulus emotion (Positive Anticipation) | 0.7124 | 0.2395 | 2.98 | 0.0029 |
| Stimulus emotion (Fear) | 0.0604 | 0.2444 | 0.25 | 0.8046 |
| Stimulus emotion (Frustration) | 0.7124 | 0.2382 | 3.01 | 0.0026 |
| Stimulus species (Human) | -2.2223 | 0.1672 | -13.29 | 0.0001 |

**12.2.Human data ECA GLMM**: There were no significant differences between the best model and the models with the added variables of PVT and stimulus species, by what these variables were dropped from the models. The largest rise in the AIC was seen whenever emotion was dropped from the model (full model AIC: 3444 *vs* model dropping emotion: 4048.5, ANOVA: *F_1_*=612.5, *p*=.0001; model with PVT and emotion AIC: 3444 *vs* model dropping emotion: 4047.1, ANOVA: *F_1_*=611.09, *p*=.0001; model with species and emotion AIC: 3442 *vs* model dropping emotion: 4047.0, ANOVA: *F_1_*=613.01, *p*=.0001; model with emotion AIC: 3442 vs null model: 4045.5, ANOVA: *F_1_*=611.52, *p*=.0001), which indicated this variable as being the only relevant one in explaining the variation in the response variable.

**12.3.Dog data PVT GLMM**: There were no significant differences between the best model and the models with the added variables of stimulus emotion and stimulus species, by what these variables were dropped from the models. Large rises in the AIC were seen whenever AOI was dropped from the models (full model AIC: 15928.3 *vs* model dropping AOI: 15953.6, ANOVA: *F_1_*=39.37, *p*=.0001; model with AOI and emotion AIC: 15927.7 *vs* model dropping AOI: 15952.1, ANOVA: *F_1_*=38.40, *p*=.0001; model with AOI and species: 15920.8 *vs* model dropping AOI: 15946.1, ANOVA: *F_1_*=39.36, *p*=.0001; model with AOI AIC: 15920.4 *vs* null model: 15944.7, ANOVA: *F_1_*=38.26, *p*=.0001), which indicated this variable as being the only one impacting the variation of PVT.

Best GLMM model for PVT as a response variable and the predictor variables AOI for the dog subset data:

| **Predictor factors** | **Estimate** | **SE** | **z** | **p** |
| --- | --- | --- | --- | --- |
| Response factor: PVT |  |  |  |  |
| Intercept | -14.109 | 0.443 | -31.84 | 0.0001 |
| AOI (frontalis) | 0.386 | 0.680 | 0.57 | 0.5699 |
| AOI (glabella) | -1.699 | 0.573 | -2.96 | 0.0031 |
| AOI (eyes) | -0.552 | 0.548 | -1.01 | 0.3136 |
| AOI (nose) | -1.635 | 0.716 | -2.28 | 0.0223 |
| AOI (cheeks) | -0.890 | 0.544 | -1.64 | 0.1020 |
| AOI (mouth) | -1.026 | 0.543 | -1.89 | 0.0587 |
| AOI (mentalis) | -3.107 | 0.718 | -4.33 | 0.0001 |

**12.4.Human vs dog data PVT GLMM**: The largest rise in the AIC was seen when the variable AOI was dropped from the model, which indicated this variable as being the most important to explain the data (best model AIC: 2984 *vs* model dropping AOI AIC: 3357, ANOVA: *F_1_*=386.99, *p*=.0001). The second largest change in AIC was when dropping the variable participant species from the model (best model AIC: 2984 *vs* model dropping participant species AIC: 3004.2, ANOVA: *F_1_*=22.23, *p*=.0001). The stimulus species and emotion also rose the AIC significantly when dropped, although a smaller increase was seen (best model AIC: 2984 *vs* model dropping stimulus species AIC: 2993.9, ANOVA: *F_1_*=11.86, *p*=.0006; and *vs* model dropping stimulus emotion AIC: 2994.5, ANOVA: *F_1_*=18.48, *p*=.0010). Stimulus species and stimulus emotion contributed equally to the model as there was no difference between the models with either variable (ANOVA: *F_1_*=6.62, *p*=.0852).

Best GLMM model for PVT as a response variable and the predictor variables AOI, participant species, stimulus species and stimulus emotion, for the data with humans and dogs as observers:

| **Predictor factors** | **Estimate** | **SE** | **z** | **p** |
| --- | --- | --- | --- | --- |
| Response factor: PVT |  |  |  |  |
| Intercept | -2.5506 | 0.1929 | -13.222 | 0.0001 |
| AOI (frontalis) | 0.3672 | 0.2564 | 1.432 | 0.1521 |
| AOI (glabella) | -1.0984 | 0.2769 | -3.967 | 0.0001 |
| AOI (eyes) | 1.4377 | 0.1789 | 8.039 | 0.0001 |
| AOI (nose) | 0.6803 | 0.2412 | 2.820 | 0.0048 |
| AOI (cheeks) | -0.7528 | 0.2498 | -3.014 | 0.0026 |
| AOI (mouth) | 0.4111 | 0.1967 | 2.091 | 0.0366 |
| AOI (mentalis) | -2.3181 | 0.4397 | -5.272 | 0.0001 |
| Stimulus emotion (happiness) | -0.5345 | 0.1562 | -3.421 | 0.0006 |
| Stimulus emotion (positive anticipation) | -0.5908 | 0.1565 | -3.774 | 0.0002 |
| Stimulus emotion (frustration) | -0.3657 | 0.1477 | -2.476 | 0.0133 |
| Stimulus emotion (fear) | -0.4315 | 0.1523 | -2.833 | 0.0046 |
| Stimulus species (human) | -0.3891 | 0.1140 | -3.415 | 0.0007 |
| Participant species (human) | 0.4751 | 0.1017 | 4.672 | 0.0001 |

**12.5.Human vs dog data PVT post-hoc Mann-Whitney tests**: Mann-Whitney, ears: *U*=58356, *p*=.001; glabella: *U*=183102, *p*=.0001; eyes: *U*=244520.5, *p*=.0001; nose: *U*=61253, *p*=.0001; cheeks: *U*=151007, *p*=.0001; mouth: *U*=215257, *p*=.0001.

**ESM 13:** P-value of the Kruskal-Wallis pairwise comparisons of the overall PVT on all AOIs for humans as observers (Bonferroni corrected), and the mean and SD for each AOI. Significant values in **bold**.

| **AOIs** | **Cheeks** | **Ears** | **Eyes** | **Frontalis** | **Glabella** | **Mentalis** | **Mouth** | **Mean** | **SD** |
| --- | --- | --- | --- | --- | --- | --- | --- | --- | --- |
| **Cheeks** |  |  |  |  |  |  |  | 0.0327 | 0.0585 |
| **Ears** | 0.8031 |  |  |  |  |  |  | 0.0240 | 0.0491 |
| **Eyes** | **0.0001** | **0.0001** |  |  |  |  |  | 0.4152 | 0.2158 |
| **Frontalis** | 0.5109 | 1.0000 | **0.0001** |  |  |  |  | 0.0272 | 0.0747 |
| **Glabella** | **0.0002** | **0.0001** | **0.0001** | **0.0001** |  |  |  | 0.0803 | 0.1222 |
| **Mentalis** | **0.0001** | **0.0001** | **0.0001** | **0.0013** | **0.0001** |  |  | 0.0055 | 0.0262 |
| **Mouth** | **0.0001** | **0.0001** | **0.0001** | **0.0001** | **0.0001** | **0.0001** |  | 0.2142 | 0.1847 |
| **Nose** | **0.0001** | **0.0001** | **0.0065** | **0.0001** | **0.0001** | **0.0001** | **0.0005** | 0.2901 | 0.1904 |

**ESM14:** See video file.

**ESM 15:** One Sample Wilcoxon tests for LVS:

**Humans looking at human stimuli**: Eyes: (One Sample Wilcoxon: happiness: V*_1_*=1364, *p*=.0001; positive anticipation: V*_1_*=1357, *p*=.0001; frustration: V*_1_*=1356, *p*=.0001; fear: V*_1_*=1247, *p*=.0001; neutral: V*_1_*=1265, *p*=.0001). Nose: (One Sample Wilcoxon: happiness: V*_1_*=1319, *p*=.0001; positive anticipation: V*_1_*=1378, *p*=.0001; frustration: V*_1_*=1368, *p*=.0001; fear: V*_1_*=1239, *p*=.0001; neutral: V*_1_*=1221, *p*=.0001). Mouth: (One Sample Wilcoxon: happiness: V*_1_*=1187, *p*=.0001; fear: V*_1_*=1074, *p*=.0001; neutral: V*_1_*=966, *p*=.002).

**Humans looking at dog stimuli:** Eyes: (One Sample Wilcoxon: happiness: V1=1275, p=.0001; positive anticipation: V1=1374, p=.0001; frustration: V1=1368, p=.0001; fear: V1=1269, p=.0001; neutral: V1=1378, p=.0001). Glabella: (One Sample Wilcoxon: happiness: V1=1131, p=.0001; positive anticipation: V1=1260, p=.0001; frustration: V1=1366, p=.0001; fear: V1=1098, p=.0001; neutral: V1=1195, p=.0001). Mouth: (One Sample Wilcoxon: happiness: V1=1077, p=.0001; positive anticipation: V1=1236, p=.0001; frustration: V1=1076, p=.0001; fear: V1=1074, p=.0001; neutral: V1=1185, p=.0001).

**Dogs looking at human stimuli:** Glabella: (One Sample Wilcoxon: positive anticipation: V*_1_*=300, *p*=.0001; neutral: V*_1_*=147, *p*=.0001). Mouth: (One Sample Wilcoxon: V*_1_*=240, *p*=.0001). Nose: (One Sample Wilcoxon: fear: V*_1_*=292, *p*=.0001; neutral: V*_1_*=240, *p*=.002).

**Dogs looking at dog stimuli:** Cheeks: (One Sample Wilcoxon: positive anticipation: V*_1_*=216, *p*=.0001; fear: V*_1_*=354, *p*=.0001). Mentalis: (One Sample Wilcoxon: neutral: V*_1_*=153, *p*=.0001).

**ESM 16:** Best GLMM model for ECA as a response variable and the predictor variables stimulus emotion.

| **Predictor factors** | **Estimate** | **SE** | **z** | **p** |
| --- | --- | --- | --- | --- |
| Response factor: ECA |  |  |  |  |
| Intercept | 0.0725 | 0.0778 | 0.933 | 0.3510 |
| Stimulus emotion (Happiness) | -0.4755 | 0.1110 | -4.283 | 0.0001 |
| Stimulus emotion (Positive Anticipation) | -1.6515 | 0.1269 | -13.014 | 0.0001 |
| Stimulus emotion (Fear) | -0.9396 | 0.1147 | -8.194 | 0.0001 |
| Stimulus emotion (Frustration) | -3.8680 | 0.2645 | -14.624 | 0.0001 |

**ESM 17**: Proportion of categorisation accuracy of the observed emotions (ECA) of human participants in human and dog stimuli. Error bars represent standard deviation:


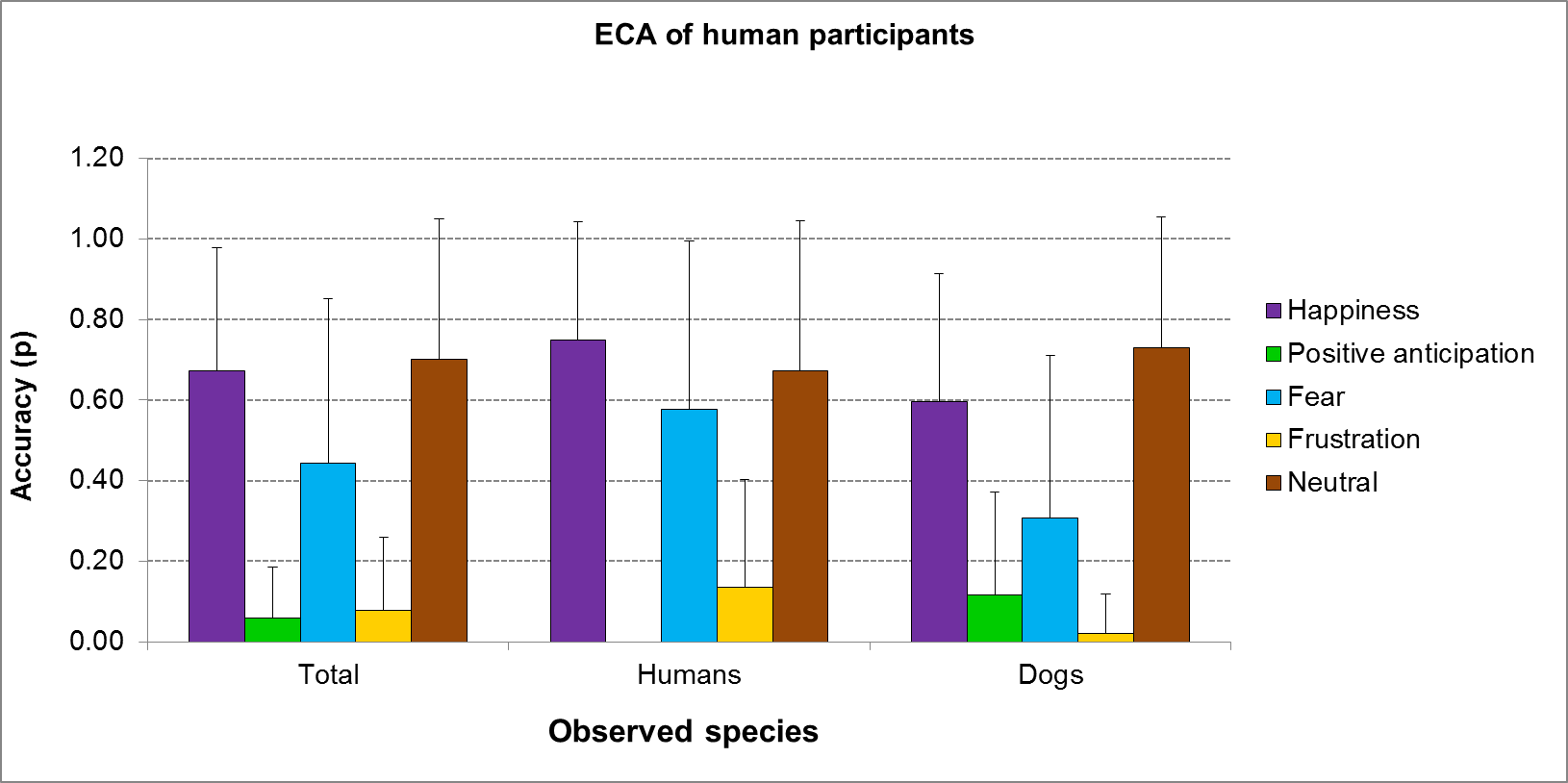


**ESM 18:**. Post-hoc Kruskal-Wallis tests between AOIs of the dog subset data.

| **AOI** | Ears | Frontalis | Glabella | Eyes | Nose | Cheeks | Mouth | Mean | SD |
| --- | --- | --- | --- | --- | --- | --- | --- | --- | --- |
| Ears |  |  |  |  |  |  |  | 0.142 | 0.250 |
| Frontalis | NS |  |  |  |  |  |  | 0.176 | 0.262 |
| Glabella | 0.0001 | 0.0001 |  |  |  |  |  | 0.041 | 0.132 |
| Eyes | 0.0256 | NS | 0.0001 |  |  |  |  | 0.072 | 0.165 |
| Nose | 0.0001 | 0.0001 | NS | 0.0014 |  |  |  | 0.040  040 | 0.137 |
| Cheeks | 0.0011 | NS | 0.0037 | NS | 0.0231 |  |  | 0.080 | 0.202 |
| Mouth | 0.0002 | NS | 0.0164 | NS | 0.0676 | NS |  | 0.090 | 0.230 |
| Mentalis | 0.0001 | 0.0001 | 0.0006 | 0.0001 | 0.0356  0356 | 0.0001 | 0.0001 | 0.016  016 | 0.100 |

**ESM 19:** Mann-Whitney tests (with Bonferroni correction) comparing humans and dogs PVT when observing conspecific and heterospecific stimuli. Significant p-values and higher means in **bold**.

| **Participant species** | | | | | | | | |
| --- | --- | --- | --- | --- | --- | --- | --- | --- |
|  |  |  |  |  | Humans |  | Dogs |  |
| **Human stimuli** | **AOI** | U | p | Bonferroni | Mean | SD | Mean | SD |
|  | Ears | 4792 | 0.0080 | 0.0036 | 0.00138 | 0.01 | 0.02586 | 0.10 |
|  | Frontalis | 29997 | 0.0330 | 0.0036 | 0.02720 | 0.08 | 0.13987 | 0.26 |
|  | Glabella | 38337 | **0.0001** | 0.0036 | 0.01679 | 0.05 | **0.01683** | 0.10 |
|  | Eyes | 59704 | **0.0001** | 0.0036 | **0.39426** | 0.23 | 0.07407 | 0.18 |
|  | Cheeks | 35004 | 0.1970 | 0.0036 | 0.03859 | 0.06 | 0.11422 | 0.24 |
|  | Nose | 61253 | **0.0001** | 0.0036 | **0.29006** | 0.20 | 0.03979 | 0.14 |
|  | Mouth | 53529 | **0.0001** | 0.0036 | **0.15334** | 0.15 | 0.07236 | 0.23 |
|  | Mentalis | 33306 | 0.7380 | 0.0036 | 0.00676 | 0.03 | 0.02466 | 0.12 |
| **Dog stimuli** | Ears  Glabella Eyes | 28376  53336  62684 | **0.0010**  **0.0001**  **0.0001** | 0.0036  0.0036  0.0036 | 0.03321  **0.14372**  **0.43608** | 0.06  0.14  0.20 | **0.18751**  0.06499  0.07073 | 0.28  0.16  0.15 |
|  | Cheeks | 40271 | **0.0001** | 0.0036 | 0.02681 | 0.05 | **0.04600** | 0.15 |
|  | Mouth | 55183 | **0.0001** | 0.0036 | **0.27498** | 0.20 | 0.10792 | 0.23 |
|  | Mentalis | 35391 | 0.0290 | 0.0036 | 0.00430 | 0.02 | 0.00704 | 0.07 |

**ESM20:** See Excel file.
